# Incidence of influenza virus infections confirmed by serology in children and adult in a suburb community, northern China, 2018‐2019 influenza season

**DOI:** 10.1111/irv.12805

**Published:** 2020-09-25

**Authors:** Cuiling Xu, Ling Liu, Binzhi Ren, Libo Dong, Shumei Zou, Weijuan Huang, Hejiang Wei, Yanhui Cheng, Jing Tang, Rongbao Gao, Lizhong Feng, Ruifu Zhang, Chaopu Yuan, Dayan Wang, Jing Chen

**Affiliations:** ^1^ Chinese National Influenza Center National Institute for Viral Disease Control and Prevention Collaboration Innovation Center for Diagnosis and Treatment of Infectious Diseases Chinese Center for Disease Control and Prevention Key Laboratory for Medical Virology National Health and Family Planning Commission Beijing China; ^2^ Shanxi Provincial Center for Disease Control and Prevention Taiyuan China; ^3^ Changzhi City Center for Disease Control and Prevention in Shanxi Province Changzhi China

**Keywords:** antibody, immunity, incidence rates, influenza virus

## Abstract

**Background:**

In mainland China, seasonal influenza disease burden at community level is unknown. The incidence rate of influenza virus infections in the community is difficult to determine due to the lack of well‐defined catchment populations of influenza‐like illness surveillance sentinel hospitals.

**Objectives:**

We established a community‐based cohort to estimate incidence of seasonal influenza infections indicated by serology and protection conferred by antibody titers against influenza infections during 2018‐2019 influenza season in northern China.

**Methods:**

We recruited participants in November 2018 and conducted follow‐up in May 2019 with collection of sera every survey. Seasonal influenza infections were indicated by a 4‐fold or greater increase of hemagglutination inhibition (HI) antibody between paired sera.

**Results:**

Two hundred and three children 5‐17 years of age and 413 adults 18‐59 years of age were followed up and provided paired sera. The overall incidence of seasonal influenza infection and incidence of A(H3N2) infection in children (31% and 17%, respectively) were significantly higher than those in adults (21% and 10%, respectively). The incidences of A(H1N1)pdm09 infection in children and adults were both about 10%, while the incidences of B/Victoria and/Yamagata infection in children and adults were from 2% to 4%. HI titers of 1:40 against A(H1N1)pdm09 and A(H3N2) viruses were associated with 63% and 75% protection against infections with the two subtypes, respectively.

**Conclusions:**

In the community, we identified considerable incidence of seasonal influenza infections. A HI titer of 1:40 could be sufficient to provide 50% protection against influenza A virus infections indicated by serology.

## 1 INTRODUCTION

Pandemic and seasonal influenza viruses have been associated with a heavy burden on morbidity and mortality worldwide.^1^, ^2^ Influenza surveillance, which provides real‐time information to inform prevention and control policy, focused on cases seeking medical care. There are some barriers to assess influenza disease burden at community level using existing surveillance platforms. The data derived from surveillance could not capture infections associated with mild symptoms or not requiring medical attention. Information on the community burden of influenza is a key to informing control. Some community‐based cohort studies have been conducted to provide important insight into the disease burden and transmission behavior of influenza.^3^, ^4^, ^5^, ^6^, ^7^, ^8^, ^9^ These studies largely took place in the United States between 1948 and 2013,^3^, ^4^, ^5^, ^6^ and recently, similar studies were undertaken in England, Hong Kong, Vietnam, etc.^7^, ^8^, ^9^ Additionally, more cohort studies were conducted to estimate disease burden of influenza during 2009‐10 pandemic influenza.^10^, ^11^

Although hospital‐based influenza‐like illness (ILI) sentinel surveillance has been established in mainland China to monitor influenza activity,^12^, ^13^ the incidence rate of influenza virus infections in the community is difficult to determine due to the lack of well‐defined catchment populations of surveillance sentinel hospitals. Very few data are available about influenza disease burden at community level in mainland China. Only several cross‐sectional serological surveys were conducted to determine the prevalence of antibodies to A(H1N1)pdm09 during the 2009 influenza pandemic.^14^, ^15^ Therefore, we established a community‐based cohort to qualify incidence of seasonal influenza infections confirmed by serology after experiencing a wintertime influenza season of 2018‐2019 in a semi‐rural community of northern China, and to estimate the protection conferred by different antibody titers against seasonal influenza infections.

## 2 METHODS

### 2.1 Recruitment and follow‐up

Our cohort study was conducted in three villages of suburb area of Changzhi City, Shanxi Province, that is located in northern China. Local residents aged 5‐59 years were recruited by doctors and nurses working at village‐level health clinics to participate in our study via face‐to‐face invitation or the invitation by phone call from mid‐November 2018 to late‐December 2019 (pre‐season survey). When the local residents agreed to participate, they were asked to complete a questionnaire including demographic, underlying medical conditions, etc. Serum samples were collected from the participants by trained nurses. The participants were followed up from mid‐May to late‐May 2019 (post‐season survey). During the post‐season survey, a short questionnaire including influenza vaccination information in 2018‐2019 influenza season and collection of sera was completed.

### 2.2 Ethics

Proxy written consent from parents or legal guardians was obtained for participants ≤17 years of age, with additional written assent from those aged 8‐17 years of age. Written consent was obtained from all adult participants. The study protocol was approved by the Institutional Review Board of Shanxi Provincial Center for Disease Control and Prevention.

### 2.3 Laboratory test

The paired sera were tested in parallel by hemagglutination inhibition (HI) assays using five representative circulating strains for each of seasonal influenza A subtypes and influenza B lineages. Four of the five representative strains used for the serological test were vaccine components recommended by WHO for the 2018‐2019 northern hemisphere influenza season, that is, A/Michigan/45/2015 (H1N1)pdm09, A/Singapore/INFIMH‐16‐0019/2016 (H3N2), B/Phuket/3073/2013 (B/Yamagata lineage), and B/Colorado/06/2017(B/Victoria lineage). Since B/Victoria lineage viruses with different genetic characteristics circulated concurrently in China, another representative strain of B/Victoria lineage with a deletion of three amino acids in hemagglutinin (HA), B/Sichuan‐Gaoxin/531/2018‐like, was selected for the serological test, besides B/Colorado/06/2017 with a deletion of two amino acids in HA. These influenza strains were provided by WHO Collaborating Centre for Reference and Research on Influenza, National Institute for Viral Disease Control and Prevention, China CDC. Influenza B viruses were treated with ether prior to use to increase sensitivity for the HI assay.^16^

The sera were treated with receptor‐destroying enzyme (RDE) at 37°C overnight to remove non‐specific inhibitors, and residual RDE was destroyed by heat inactivation at 56°C for 30 minutes. The standardized turkey or guinea‐pig red blood cells were added to the sera to remove non‐specific agglutinins, and the adsorbed sera without disturbing the packed RBCs were transferred. The HI assay was carried out according to standard methods described in the WHO guideline.^17^ Sera were tested in serial doubling dilutions from an initial dilution of 1:10 to endpoint dilution of 1:1280 by HI assays using standard methods. Antibody titers <1:10 were imputed as 1:5, and antibody titers ≥1:1280 were imputed as 1:1280.

### 2.4 Statistical analysis

Hemagglutination inhibition antibody titers of paired sera were compared to determine serologic evidence of infections of influenza viruses, indicated by a 4‐fold or greater increase in antibody titers between pre‐season and post‐season sera with an antibody titer of 1:40 or more in post‐season sera. We used Poisson regression model to estimate the incidence rates of influenza virus infections indicated by serology in children aged 5‐17 years and adults aged 18‐59 years, respectively. We used a logistic mixed‐effect regression model to estimate the association of influenza infections indicated by serology with age‐groups and other factors. We used logistic regression to estimate the relationship between protection against influenza infections and antibody titers.^18^ The protection rate at different antibody titer was calculated as the odd ratio (OR) reduction compared with the OR at an HI titer <1:10. We compared medians of pre‐season HI antibody titers between children and adults, between participants infected and not infected with seasonal influenza viruses, using Wilcoxon signed‐rank test after log transformation.

## 3 RESULTS

Our study recruited 228 children aged 5‐17 years and 457 adults aged 18‐59 years from mid‐November to late‐November, 2018. Two hundred and three children (89%) and 413 adults (90%) were followed up from mid‐May to late‐May 2019 and provided pre‐season and post‐season sera. The characteristics of subjects participating in pre‐season and post‐season surveys were similar (Table 1). 31% of participants followed up were male. 10% of participants followed up had tertiary education or above. None of the participants received seasonal influenza vaccine during the 2018‐2019 influenza season. Of 616 participants who were followed up, 426 participants clustered in 192 households. There were significantly higher proportions of children 5‐17 years of age, students, and male participants among the participants from household clusters than among those not from household clusters (Table 2).

**TABLE 1:** Characteristics of study participants completing pre‐season survey and both of pre‐season and post‐season survey

Characteristics	Pre‐season	Post‐season
Age, y		
5‐9	93 (14)	80 (13)
10‐14	106 (15)	96 (16)
15‐17	29 (4)	27 (4)
18‐29	55 (8)	47 (8)
30‐44	225 (33)	202 (33)
45‐59	177 (26)	164 (27)
Gender, Male	232 (34)	190 (31)
Ethnic, Han	680 (99)	611 (99)
Occupation		
Students	209 (30)	208 (34)
Farmer and worker	290 (42)	263 (43)
Professional and technical staff	134 (20)	118 (19)
Administrative and managerial staff	21 (3)	21 (3)
Education		
Primary or below	210 (31)	207 (34)
Secondary	372 (54)	338 (55)
Tertiary or above	65 (9)	59 (10)
Self‐perceiving good health status	521 (76)	465 (75)
Influenza vaccination in 2018‐2019^a^	—	0 (0)
No. of households		
With 1 participant	205 (30)	190 (31)
With 2 participants	318 (46)	300 (49)
With 3 participants	150 (22)	126 (20)
With 4 participants	12 (2)	0 (0)

^a^ The information on influenza vaccination of participants in 2018‐2019 was obtained in post‐season survey.

**TABLE 2:** Characteristics of study participants followed up with and without household cluster in post‐season survey

Characteristics	Participants from household clusters	Participants not from household clusters	*P* values
Age, y			
5‐17	51 (27)	152 (36)	<.05
18‐59	139 (73)	274 (64)	
Gender, Male	43 (23)	147 (35)	<.01
Ethnic, Han	188 (99)	423 (99)	1.00
Occupation			
Students	51 (27)	157 (37)	<.001
Farmer and worker	104 (55)	159 (37)	
Professional and technical staff	26 (14)	92 (22)	
Administrative and managerial staff	8 (4)	13 (3)	
Education			
Primary or below	63 (33)	144 (34)	.72
Secondary	103 (54)	235 (55)	
Tertiary or above	21 (11)	38 (9)	
Self‐perceiving good health status	143 (75)	322 (76)	1.00
Influenza vaccination in 2018‐2019^a^	0 (0)	0 (0)	—

^a^ The information on influenza vaccination of participants in 2018‐2019 was obtained in post‐season survey.

**FIGURE 1:**
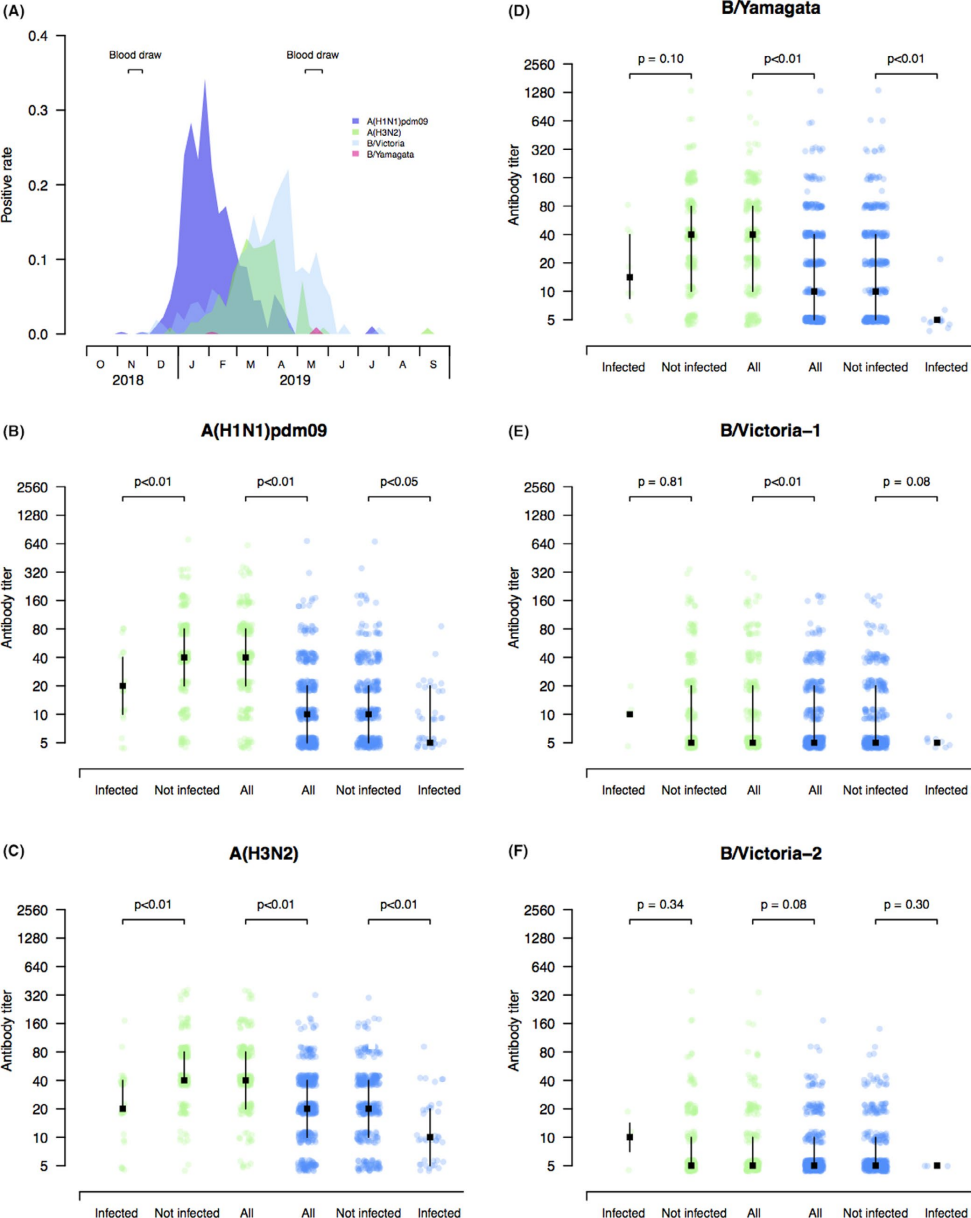
A, Timeline of the study and influenza activity in Shanxi Province. B‐F, Pre‐season HI antibody titer measurements (points), and medians and interquartile range against the vaccine strains of A(H1N1)pdm09, A(H3N2), and B/Yamagata, as well as B/Colorado/06/2017‐like (B/Victoria‐1) and B/Sichuan‐Gaoxin/531/2018‐like (B/Victoria‐2) virus by infection status of individuals in children and adults. The *y*‐axis indicates the pre‐season HI antibody titer measurements. Individuals in children (green dots) and adults (blue dots) were separated. Analyses were performed for all individuals as well as separately for individuals infected and not infected with each influenza strain used for serological test in children and adults, respectively. Two‐tailed Wilcoxon rank‐sum tests were used to compare pre‐season log‐transformed HI antibody titers between groups

During the 2018‐2019 influenza season, influenza activity increased since December 2018 and decreased to a low level until mid‐June 2019 in Shanxi Province (Figure 1A). During this season, Epidemics of influenza A(H1N1)pdm09, A(H3N2), and B/Victoria virus occurred successively, which peaked there at January, March, and April 2019, respectively.

Totally, we confirmed 151 seasonal influenza infections indicated by serology during the study period, including 61 influenza A(H1N1)pdm09, 74 A(H3N2), 17 B/Victoria, and 20 B/Yamagata. Of those infections, 19 (13%) had infections with two or more influenza subtypes or lineages in the same season, including 6 children aged 5‐17 years. The incidences of seasonal influenza viruses in children and adults during the 2018‐2019 influenza season are shown in Table 3. During the influenza season, the overall incidence of seasonal influenza virus in children (31%; 95% confidence interval [CI], 24%‐40%) was significantly higher than that in adult (21%; 95% CI, 17%‐25%). The significant difference in the incidence of influenza A(H3N2) virus between children (17%; 95% CI, 12%‐24%) and adults (9%; 95% CI, 6%‐12%) contributed to the difference in the overall incidence of infections between the two age‐groups. The incidences of influenza A(H1N1)pdm09 in children and adults were approximately 10%, while the risks of infection with B/Victoria and B/Yamagata in children and adults were 2%‐4%. Gender, occupation, and self‐perceiving health status were not associated with serological evidence of seasonal influenza infections.

**TABLE 3:** Incidence of seasonal influenza infections confirmed by serology by subtype and lineage during the 2018‐2019 influenza season

Influenza subtype/lineage	5‐17 y		18‐59 y		*P* value^a^
	Incidence	95% CI	Incidence	95% CI	
A(H1N1)pdm09	0.10	0.06‐0.15	0.10	0.07‐0.13	.51
A(H3N2)	0.17	0.12‐0.24	0.09	0.06‐0.12	<.01
B/Victoria	0.03	0.02‐0.07	0.02	0.01‐0.05	.55
B/Yamagata	0.04	0.02‐0.08	0.03	0.02‐0.05	.67
Overall	0.31	0.24‐0.40	0.21	0.17‐0.25	<.05

^a^ A logistic mixed‐effect regression model used to estimate the association of influenza infections indicated by serology between age‐groups.

**FIGURE 2:**
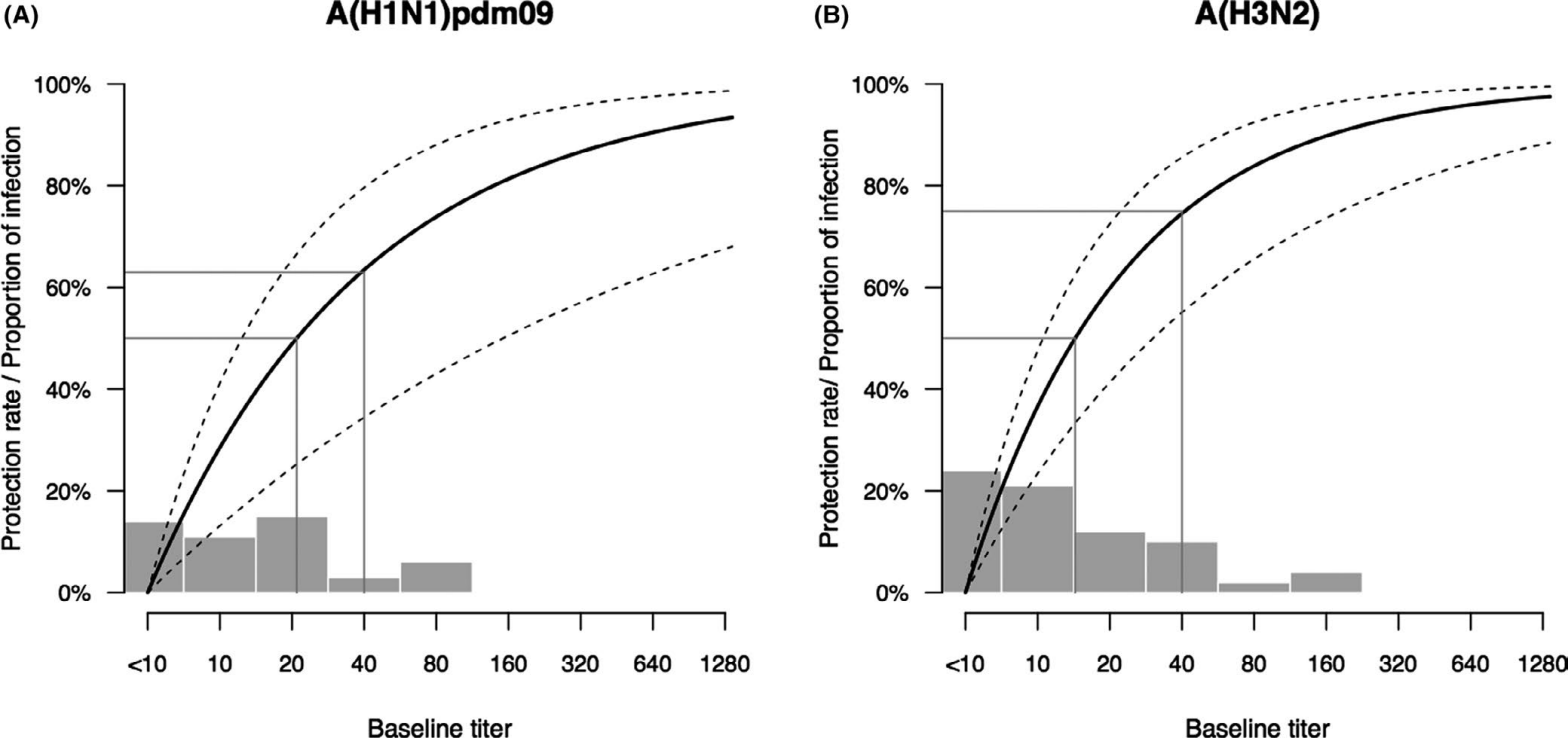
Correlation between reciprocal antibody titers and protection against seasonal influenza infection confirmed by serology. A, Protection among participants with A(H1N1)pdm09 infection associated with reciprocal HI titers against A(H1N1)pdm09 virus. B, Protection among participants with A(H3N2) infection associated with reciprocal HI titers against A(H3N2) virus. Reciprocal antibody titers corresponding to 50% protection and protection rate corresponding to a antibody titers of 1:40 are indicated with gray lines. The gray bars show the proportion of participants having a certain HI antibody titer. The bars group individuals between the antibody titers covered by the bars on the *x*‐axis

The higher pre‐season HI antibody titers against influenza A (H1N1) pdm09 and influenza A (H3N2) viruses in children and adults were associated with a reduced risk of infection of the two subtypes indicated by serology (*P* < .05). HI titers of 1:40 against A(H1N1)pdm09 and A(H3N2) viruses were associated with 63% (95% CI, 34%‐80%) and 75% (95% CI, 55%‐86%) protection against infections with the two subtypes indicated by serology, respectively, while a HI titers of 1:20 against A(H1N1)pdm09 and A(H3N2) corresponded to 49% (95% CI, 25%‐65%) and 60% (95% CI, 41%‐72%) protection against serological evidence of infections with the two subtypes (Figure 2A,B). We estimated that the level of HI titers against seasonal A(H1N1) and A(H3N2) that were associated with 50% protection against serologically confirmed seasonal A (H1N1) and A(H3N2) virus infection was 1:21 (95% CI, 1:12‐1:152) and 1:14 (95% CI, 1:11‐1:30), respectively. We did not identify the association of pre‐season HI antibody titers and serological evidence of infections with B/Victoria and B/Yamagata, possibly due to a low proportion of infections with the two lineages of influenza B.

We found the medians of pre‐season HI antibody titers against the representative circulating strains of influenza A(H1N1)pdm09, A(H3N2), and B/Yamagata in children were significantly higher than those in adults (Figure 1B‐D). There was also a significant difference in pre‐season HI antibody titer against B/Colorado/06/2017(B/Victoria lineage) between children and adults, while the difference in pre‐season HI antibody titer against B/Sichuan‐Gaoxin/531/2018‐like virus (B/Victoria lineage) between children and adults was not identified (Figure 1E,F).

## 4 DISCUSSION

We established a cohort of children and adults in an unvaccinated community and collected their paired sera before and after 2018‐2019 influenza season to enable inference on incidence of seasonal influenza infections. Our study showed that seasonal influenza infections were common in unvaccinated children and adults. We identified the overall risks of infection with seasonal influenza viruses were 31% and 21% in children aged 5‐17 years and adults aged 18‐59 years, respectively.

There were higher risks of infections with A(H1N1)pdm09 and A(H3N2) in both children and adults, compared with the risks of infections with B/Victoria and B/Yamagata. However, the risk of infection with A(H1N1)pdm09 between children and adults is similar, and the risk of infection with A(H3N2) in children was significantly higher than that in adults. The difference could be related to degree of coincidence between A(H1N1)pdm09 and A(H3N2) epidemic and school winter holiday. According to surveillance data of influenza activity in Shanxi Province, influenza A(H1N1)pdm09 activity peaked in January 2019, while primary and middle schools started their winter vacation since early January of this year. Influenza A(H3N2) activity peaked in March 2019, while primary and middle schools ended their winter holiday at the end of February of this year. Many previous studies have reported that school‐aged children have the highest attack rate of seasonal and pandemic influenza because children within schools could increase potential for transmission of influenza virus.^7^, ^8^, ^10^, ^14^, ^19^

Our study found HI titers of 1:40 against A(H1N1)pdm09 and A(H3N2) were correlated with approximately 63% and 70% protection against infections with A(H1N1)pdm09 and A(H3N2) indicated by serology. While data from experimental challenge studies, clinical trial and observational studies in community settings showed that an HI titer of 1:40 was associated with approximately 50% or less protection against clinical influenza or virologically confirmed influenza infection.^20^, ^21^, ^22^, ^23^ One possible explanation is the difference in definition of influenza infection between our study and the previous studies. It is known that influenza infection indicated by serology includes symptomatic and asymptomatic infection. The asymptomatic influenza infection could be related to less viral replication and shedding during infection, compared with clinically or virologically confirmed infection. As reported in the previous community‐based study, only 11%‐25% of serologically confirmed infections had PCR‐confirmed illness.^7^, ^8^ The other possible explanation is that influenza HI antibody of all subjects mainly originated from natural infection of virus in our study while in other studies all or a part of subjects could have antibody induced by inactivated or attenuated virus used for vaccine.^23^

As observed in the previous prospective studies, our study identified multiple infections with different influenza subtype/lineage in the same season, and they could occur in children and adults.^8^, ^24^ So far, the scientific significance of the phenomenon is unclear.

We observed that pre‐season HI antibody titers against four vaccine strains in children were all higher than those in adults, while there was no a difference in HI antibody titer against another B/Victoria virus (B/Sichuan‐Gaoxin/531/2018‐like) between children and adults. The possible explanation for the observation is that these vaccine strain‐like viruses have circulated moderately or widely in the local community for a period of time so as to bring about higher infection‐induced antibody levels against these viruses in school‐aged children prior to the 2018‐2019 influenza, while B/Sichuan‐Gaoxin/531/2018‐like virus could circulate at a very low level during the same period. According to the Chinese influenza surveillance report, the vast majority or a substantial proportion of the isolated viruses of each subtype/lineage were antigenically closely related to their corresponding vaccine viruses prior to the 2018‐2019 influenza season.^25^ There could be a small proportion of B/Sichuan‐Gaoxin/531/2018‐like viruses among the isolated B/Victoria lineage viruses in Shanxi Province prior to the 2018‐2019 influenza season.

There are a number of limitations in our study. When we conducted the post‐season survey, the 2018‐2019 influenza season, especially the epidemic wave of B/Victoria lineage virus did not end. So our study could underestimate the incidence of the B/Victoria lineage virus infection and overall incidence of influenza virus infections indicated by serology. Although we estimated the association of influenza infections with risk factors using a logistic mixed‐effect regression that allow for household clusters, we did not use a model accounting for random effects of the household clusters when we estimated the relationship between protection against influenza infections and HI antibody titers. Our study use a 4‐fold rise or more in HI antibody titers in paired sera plus a antibody titer of 1:40 or more in post‐season sera as the case definition of serological evidence of influenza infection. The strict case definition could lead to underestimate the incidence of infections indicated by serology. A study showed the case definition based on 4‐fold rise or greater may have the potential of underestimation of community attack rates.^26^ Finally, due to limited research resource, we cannot undertake surveillance for clinical illness of subjects. So we cannot estimate the protection rate of HI antibody titers against clinical illness.

In conclusion, we found substantial incidence of seasonal influenza infections in the unvaccinated community during the 2018‐2019 influenza season. The school‐aged children had higher overall incidence of seasonal influenza infections and higher incidence of A(H3N2) infection than adults aged 18‐59 years. We demonstrated that a HI titer of 1:40 was sufficient to provide 50% protection against influenza A infections indicated by serology.

## CONFLICT OF INTEREST

The authors report no other potential competing interests.

## AUTHOR CONTRIBUTIONS

**Cuiling Xu:** Conceptualization (equal); Data curation (equal); Investigation (equal); Methodology (equal); Writing‐review & editing (equal). **Ling Liu:** Funding acquisition (equal); Investigation (equal); Validation (equal). **Binzhi Ren:** Investigation (equal); Methodology (equal); Supervision (equal). **Libo Dong:** Methodology (equal); Validation (equal). **Shumei Zou:** Investigation (equal); Validation (equal). **Weijuan Huang:** Methodology (equal); Supervision (equal). **Hejiang Wei:** Investigation (equal); Validation (equal). **Yanhui Cheng:** Investigation (equal). **Jing Tang:** Investigation (equal). **Rongbao Gao:** Investigation (equal). **Lizhong Feng:** Project administration (equal); Resources (equal). **Ruifu Zhang:** Conceptualization (equal); Project administration (equal); Resources (equal). **Chaopu Yuan:** Project administration (equal); Resources (equal). **Dayan Wang:** Funding acquisition (equal); Methodology (equal); Supervision (equal). **Jing Chen:** Project administration (equal); Resources (equal).

## Data Availability

The datasets in our study are available from the first author and correspondence author.
